# Clinical applications and prospects of PET imaging in patients with IDH-mutant gliomas

**DOI:** 10.1007/s11060-022-04218-x

**Published:** 2022-12-29

**Authors:** Michael M. Wollring, Jan-Michael Werner, Garry Ceccon, Philipp Lohmann, Christian P. Filss, Gereon R. Fink, Karl-Josef Langen, Norbert Galldiks

**Affiliations:** 1 Institute of Neuroscience and Medicine (INM-3, -4), Research Center Juelich, Leo-Brandt-St., 52425 Juelich, Germany; 2 Present Address: Department of Neurology, Faculty of Medicine and University Hospital Cologne, University of Cologne, Kerpener St. 62, 50937 Cologne, Germany; 3 Department of Nuclear Medicine, University Hospital Aachen, Aachen, Germany; 4 Center of Integrated Oncology (CIO), Universities of Aachen, Bonn, Cologne, and Duesseldorf, Cologne, Germany

**Keywords:** Amino acid PET, Astrocytoma, Oligodendroglioma, Non-enhancing gliomas, IDH inhibitors

## Abstract

PET imaging using radiolabeled amino acids in addition to MRI has become a valuable diagnostic tool in the clinical management of patients with brain tumors. This review provides a comprehensive overview of PET studies in glioma patients with a mutation in the isocitrate dehydrogenase gene (IDH). A considerable fraction of these tumors typically show no contrast enhancement on MRI, especially when classified as grade 2 according to the World Health Organization classification of Central Nervous System tumors. Major diagnostic challenges in this situation are differential diagnosis, target definition for diagnostic biopsies, delineation of glioma extent for treatment planning, differentiation of treatment-related changes from tumor progression, and the evaluation of response to alkylating agents. The main focus of this review is the role of amino acid PET in this setting. Furthermore, in light of clinical trials using IDH inhibitors targeting the mutated IDH enzyme for treating patients with IDH-mutant gliomas, we also aim to give an outlook on PET probes specifically targeting the IDH mutation, which appear potentially helpful for response assessment.

## Introduction

With the fifth edition of the World Health Organization (WHO) classification of tumors of the Central Nervous System (CNS) from 2021, gliomas with isocitrate dehydrogenase (IDH) mutations are classified either as diffuse astrocytoma of the WHO CNS grade 2–4, or as oligodendroglioma of the WHO CNS grade 2–3, if a 1p/19q co-deletion is additionally present [1].

At initial diagnosis, during treatment, and at follow-up of patients with IDH-mutant gliomas, anatomical MRI is the imaging modality of choice given its widespread availability and excellent spatial resolution [2]. In contrast, the specificity of this technique for neoplastic tissue is low, and a disruption of the blood–brain barrier indicated by contrast enhancement is not limited to neoplastic tissue [3–9]. Besides, both contrast enhancement and signal changes in fluid-attenuated inversion recovery (FLAIR) and T2-weighted images may be induced by inflammation, ischemia, or reactive changes following neurooncological therapy [8, 10–12]. Of note, IDH-mutant gliomas of the WHO CNS grade 2 are mostly non-enhancing on MRI and usually evaluated solely based on a hyperintense signal extension of FLAIR- or T2-weighted MRI sequences. Furthermore, even a subgroup of IDH-mutant gliomas of the WHO CNS grades 3 or 4 may also be non-enhancing [13].

These limitations of anatomical MRI may negatively affect a reliable delineation of the spatial extent of non-enhancing glial tumors for planning of a diagnostic biopsy, tumor resection, and other local treatment options such as radiotherapy including the assessment of response [9, 14]. In particular, overlooking the most malignant tumor parts may lead to both inaccurate diagnosis and grading of tumors characterized according to the WHO CNS classification.

To overcome these diagnostic challenges, PET imaging using radiolabeled amino acid tracers has become increasingly important over the past decades [9, 14–16]. Furthermore, the clinical value of this group of tracers, besides of anatomical MRI, has been recommended by the PET task force of the Response Assessment in Neuro-Oncology (RANO) Working Group for the diagnostic management of patients with either gliomas or brain metastases [17, 18].

This review summarizes the value of amino acid PET for the most relevant clinical indications in patients with IDH-mutant gliomas, with particular emphasis on non-enhancing tumors.

## Methods

A PubMed search using the search terms “PET”, “glioma”, “amino acid”, “MET”, "FET", “FDOPA”, “isocitrate dehydrogenase”, “IDH”, “non-enhancing glioma”, “radiotherapy”, “radiation necrosis”, “pseudoprogression”, “tumor extent”, “response assessment”, “treatment-related changes”, ”immunotherapy”, and combinations thereof was performed until July 2022.

## Amino acid PET

Different PET tracers have been evaluated in patients with gliomas and other brain tumors to visualize and quantify multiple metabolic properties such as glucose consumption, amino acid transport, proliferation, hypoxia, blood flow, and angiogenesis [19]. This section focuses on PET imaging using radiolabeled amino acids, which are increasingly used in the diagnostic management of patients with glioma, especially in Europe.

The most used amino acid tracers for PET imaging to date are O-(2-[^18^F]-fluoroethyl)-L-tyrosine (FET), [^11^C]-methyl-L-methionine (MET), and 3,4-dihydroxy-6-[^18^F]-fluoro-L-phenylalanine (FDOPA). Their uptake is facilitated by large neutral amino acid transporters of the L‑type (LAT) in gliomas and brain metastases (i.e., subtypes LAT1 and LAT2), which are regularly overexpressed in both brain tumor types [9, 20–23]. Most early amino acid PET studies were performed using MET, but the short half-life of 20 min imposes logistical challenges, necessitating an onsite cyclotron [24, 25]. The advent of ^18^F-labeled tracers with a considerably longer half-life (110 min) such as FET and FDOPA allows transport to other facilities and has led to the replacement of MET predominantly by FET, especially in Europe [9]. Notably, the physiological uptake of FDOPA in the striatum may hamper its use in evaluating tumor extent [9, 26].

In recent years, the synthetic amino acid analog anti-1-amino-3-[^18^F]fluo rocyclobutane-1-carboxylic acid (Fluciclovine) has gained clinical interest particularly for imaging of primary and secondary brain tumors. Intratumoral transport of Fluciclovine seems to be mediated by LAT1, but predominantly by the neutral alanine, serine, cysteine transporter 2 (ASCT2), another neutral amino acid transporter [27].

## Most important clinical applications

### Characterization of newly diagnosed non-enhancing brain lesions for differential diagnosis using amino acid PET

Brain lesions presenting hyperintense FLAIR signal alterations on anatomical MRI without concomitant contrast enhancement may frequently suggest an IDH-mutant non-enhancing glioma. On the other hand, these lesions constitute a heterogeneous group of diseases including non-neoplastic lesions such as cerebral hematoma, ischemic lesions, inflammatory or infectious processes, and even malignant gliomas without disrupted blood–brain barrier. False interpretation of these findings may result in necessary treatment being deferred or unnecessarily indicated.

Amino acid PET has a sensitivity of more than 90% to detect gliomas [28–30], but grade 2 gliomas characterized according to older WHO classifications of tumors of the CNS [31, 32] exhibit increased tracer uptake only in 70–80% [28–30]. The remaining gliomas are not avid on amino acid PET (i.e., no increased uptake compared to the unaffected brain tissue). Of note, a subgroup of patients without FET uptake in brain lesions with MRI findings suspicious for low-grade gliomas (i.e., hyperintense T2/FLAIR lesion without contrast enhancement) may even show photopenic defects on FET PET (i.e., FET uptake visually lower than the healthy background uptake) and harbor malignant gliomas [33]. This phenomenon has also been described for the radiolabeled amino acids MET and FDOPA [34].

Regarding the diagnostic performance of FET PET for differential diagnosis, a meta-analysis evaluating 13 studies with a total of 462 patients reported a specificity of 76% and a sensitivity of 82% for differentiating primary brain tumors from non-tumoral lesions [35]. A subsequent FET PET study including 174 patients (n = 73 patients with WHO CNS grade 2 gliomas, 75% of these without contrast enhancement) at initial diagnosis of cerebral lesions suggestive of glioma yielded a higher specificity (92%) and a positive predictive value for glioma tissue of 98% using a maximum tumor-to-brain ratio of 2.5 [36].

For differential diagnosis within the subgroup of gliomas, patients with oligodendroglioma characterized by an IDH mutation and a 1p/19q co-deletion [1] frequently exhibit a considerably higher amino acid uptake than patients with IDH-mutant astrocytomas [37]. For example, a more recent MET PET study by Kim and co-workers reported that in 74 patients with IDH-mutant gliomas of the WHO CNS grade 2 or 3, oligodendrogliomas had significantly higher average median tumor-to-brain ratios than astrocytomas (2.90 vs. 1.40; P < 0.001), but not higher than in patients with IDH-wildtype glioma (n = 70) (averaged median tumor-to-brain ratio, 3.35) [38].

In summary, amino acid PET may add differential diagnostic information in patients with brain lesions suggestive of non-enhancing glioma. Of note, available studies do not primarily focus on patients with IDH-mutant gliomas. Thus, the impact of this genetic alteration on the diagnostic performance of amino acid PET warrants further investigation.

### Target definition for diagnostic biopsy and delineation of glioma extent for treatment planning using amino acid PET

**Fig. 1:**
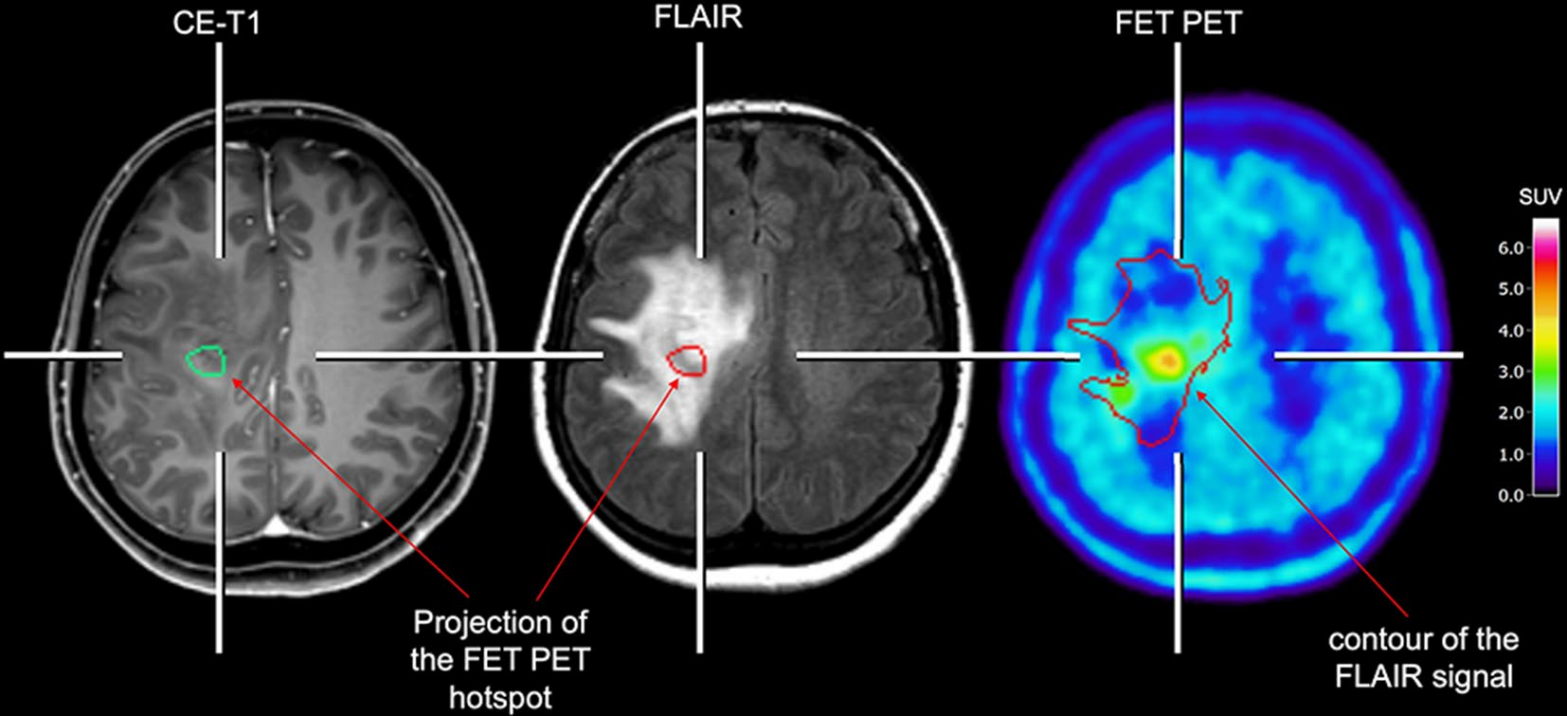
Contrast-enhanced MRI and FET PET of a 49-year-old female patient with a large FLAIR-hyperintense lesion without contrast enhancement in the right central cortex suggesting a glioma. Due to the widespread FLAIR signal, a definite biopsy target is lacking. The additional FET PET scan shows a localized area with pathologically increased metabolic activity (maximum tumor-to-brain ratio, 3.3), considerably smaller than the hyperintense FLAIR signal (red contour), and thereby offers a target for diagnostic biopsy. After FET PET-guided stereotactic biopsy of the metabolically active lesion, neuropathological evaluation of the obtained tissue revealed an IDH-mutant astrocytoma, WHO CNS grade 2

Particularly in patients with non-enhancing gliomas, it is difficult to identify the biopsy target including the most malignant tumor parts using anatomical MRI [39–41], especially when a widespread T2/FLAIR signal on MRI is present [42]. For PET, several studies have correlated histomolecular findings obtained from tissue specimens with imaging findings on amino acid PET, predominantly using the tracers FET and MET, and provided evidence that this technique detects the solid mass of gliomas including most malignant parts and the metabolically active tumor more reliably than conventional MRI [43–49] Fig. 1. Therefore, amino acid PET appears to be a highly valuable tool for target definition. In addition, combining amino acid PET with advanced MRI techniques such as diffusion- or perfusion-weighted MR imaging may further improve target definition for diagnostic biopsy planning [47, 50]. Initial studies suggest that also the synthetic amino acid analog Fluciclovine accumulates in non-enhancing gliomas and identifies infiltrating tumor areas without contrast enhancement on MRI [51, 52]. Considering the significantly higher tumor-to-brain contrast of Fluciclovine compared to other amino acid tracers [53], this tracer may be of additional value for the delineation of tumor extent and target definition in this group of patients.

Using dynamic FET PET acquisition, additional imaging parameters derived from time-activity curves such as time-to-peak values (i.e., time from tracer injection to maximum tracer uptake) or quantitative approaches to characterize time-activity curve patterns (e.g., calculation of slope) can be obtained [54]. For instance, the information derived from dynamic FET PET parameters may also help identify most malignant tumor parts in suspected gliomas, thus offering an additional tool to define the biopsy target [39–41].

Furthermore, accurate delineation of tumor extent to ensure a maximal resection is also of particular interest in patients with IDH-mutant gliomas, as even minimal tumor remnants after surgery may negatively impact overall survival [55]. A recent study by Ninatti et al. compared the additional value of preoperative MET PET for delineating tumor extent in patients with IDH-mutant gliomas (i.e., oligodendrogliomas and astrocytomas of the WHO CNS grade 2 or 3) with anatomical MRI alone [56]. In that study, MET PET improved the target volume for surgical resection in 28 of 153 patients (25%). Moreover, in patients with IDH-mutant astrocytomas, higher maximum tumor-to-brain ratios on preoperative MET PET were independent predictors of shorter progression-free survival [56].

### Differentiation of treatment-related changes from glioma progression using amino acid PET

**Fig. 2:**
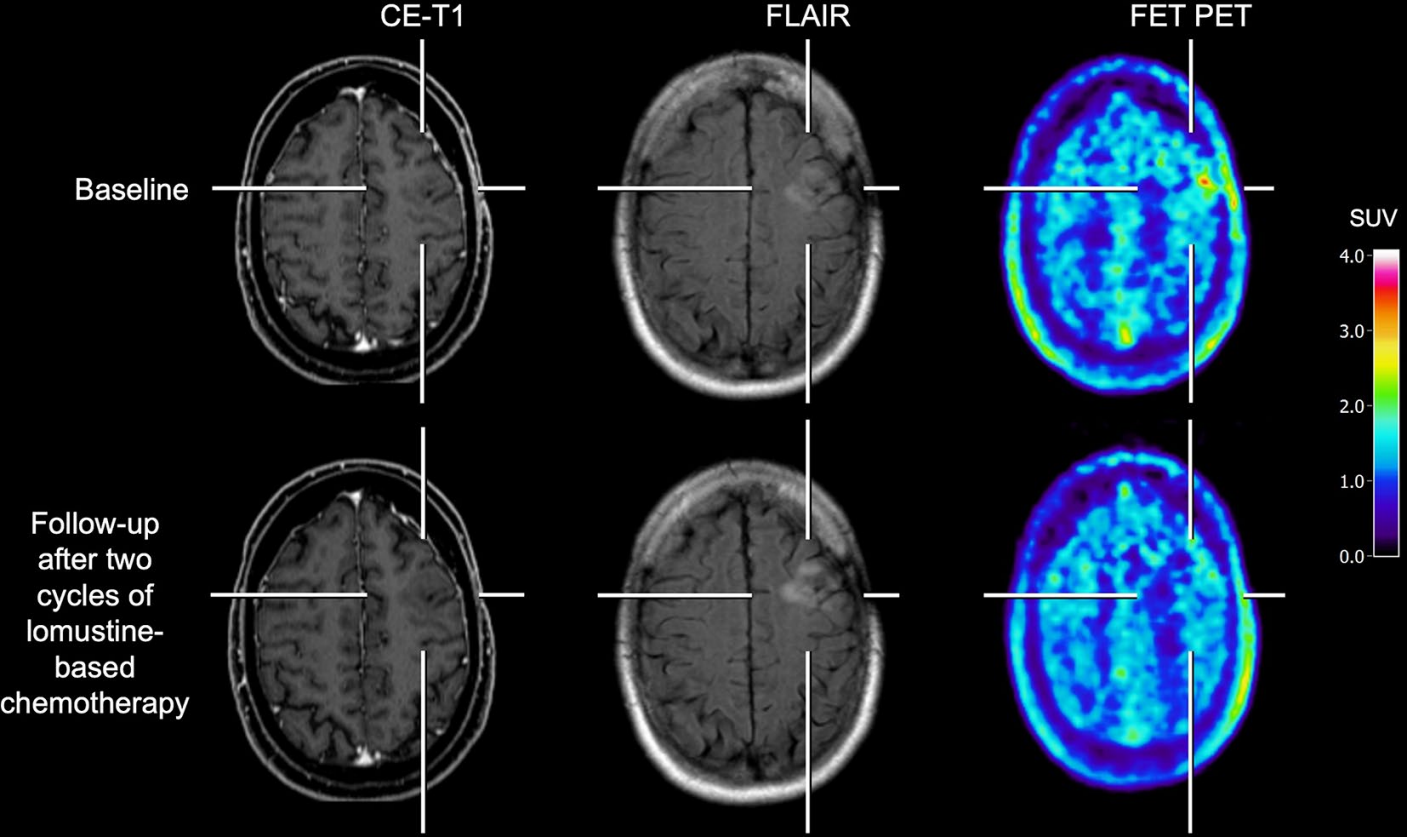
Contrast-enhanced MRI and FET PET of a 52-year-old male patient with a left frontal IDH-mutant, 1p/19q-codeleted oligodendroglioma of the WHO CNS grade 2 after resection and radiotherapy (top row). The corresponding FET PET scan shows residual metabolic activity (maximum tumor-to-brain ratio, 2.1). After two cycles of adjuvant chemotherapy with procarbazine and lomustine, the follow-up MRI (bottom row) shows an increasing FLAIR signal alteration without contrast enhancement, suggesting tumor progression. In contrast, the follow-up FET PET shows no increased metabolic activity, indicating a metabolic response. During adjuvant chemotherapy, the hyperintense FLAIR signal regressed partially, and the patient was free of tumor progression for more than two years

The differentiation of treatment-related changes such as pseudoprogression or radiation necrosis from tumor progression is of utmost importance in clinical routine. Especially amino acid PET using FET [57–65] or FDOPA [66–68] achieved a high diagnostic accuracy for differentiating treatment-related changes from tumor progression in glioma patients Fig. 2. Of note, these studies have been performed primarily on IDH-wildtype glioblastoma patients. In more recent studies, the diagnostic performance of amino acid PET for this indication has also been evaluated in patients with IDH-mutant gliomas. In a study including 127 patients (48% of patients had IDH-mutant gliomas), Maurer and colleagues reported that the combined analysis of static and dynamic FET PET parameters achieved an overall accuracy of 81% for the differentiation of treatment-related changes from tumor progression [69]. Interestingly, a subgroup analysis suggested that the diagnostic accuracy was only 67% in patients with IDH-mutant gliomas compared to 91% in IDH-wildtype glioma patients [69]. On the other hand, in subsequent studies the latter finding could be confirmed only partially [70–72], indicating that further studies in this subgroup of patients with IDH-mutant gliomas are warranted.

### Assessment of response to alkylating chemotherapy using amino acid PET

Two studies evaluated the response to temozolomide chemotherapy in patients with previously untreated WHO grade 2 gliomas classified according to older WHO classifications [31, 32] using serial MET PET or FET PET compared to anatomical MRI [73, 74]. In one of these studies, the IDH mutational status was partially available (67% of patients had a confirmed IDH mutation) [74]. A metabolic response, defined as a decrease of metabolically active tumor volume of more than 10% was observed in 8 of 11 patients (73%), and in 25 of 33 patients (76%), respectively [73, 74]. Notably, MET and FET PET detected treatment response significantly earlier than FLAIR signal changes on MRI. Moreover, a reduced metabolic tumor volume was associated with a significantly longer progression-free survival and an improved seizure control [74].

In a more recent study by Suchorska et al. [75] FET PET and MRI were used to assess response to temozolomide or lomustine-based regimens in 61 patients with non-enhancing gliomas, including 19 patients with IDH-mutant, 1p/19q-non-codeleted gliomas (31%), and 30 IDH-mutant and 1p/19q-codeleted gliomas (49%). Metabolic response, defined as any decrease of the metabolic tumor volume, was observed in 34 patients (range of decrease relative to baseline, 10–25%). These patients had a significantly longer time-to-treatment failure than patients with stable or increasing metabolic tumor volumes (median time, 78.5 vs. 24.1 months; P = 0.001). On the other hand, signal changes on T2-weighted MRI did not correlate with the patients’ survival.

### Future prospects

#### PET imaging of the IDH mutation

In patients with IDH-mutant gliomas, the efficacy of novel therapies targeting the mutant IDH enzyme using oral IDH1-inhibitors (e.g., ivosidenib), IDH2-inhibitors such as endasidenib, inhibitors of both IDH1 and IDH2 mutations (e.g., vorasidenib), and vaccines targeting the IDH1 (R132H) neoepitope are currently under investigation. Initial phase I clinical trials suggested promising antitumoral activity [76–78]. At initial diagnosis, neuropathological diagnostics, including genomic sequencing, is currently the method of choice for detecting an IDH mutation. During follow-up, quantification of changes of IDH expression levels in patients undergoing these treatment options using neuropathological techniques always requires the invasive tissue removal. Alternatively, the use of proton MR spectroscopy for the non-invasive evaluation of signal changes of the oncometabolite 2-hydroxyglutarate related to IDH mutations is an option for response assessment, but this technique is highly prone to susceptibility artefacts due to bone, hemorrhage, calcifications, or surgical material and may even be false-positive in 20% of patients with newly diagnosed IDH-wildtype glioblastoma [79, 80].

Regarding PET imaging of IDH mutations, novel PET tracers such as radiolabeled triazinediamine or butyl-phenyl sulfonamide analogs, and the radiolabeled IDH1 inhibitor AGI-5198 may be valuable candidates [81–83]. Furthermore, in an animal study, Koyaso and colleagues observed that the uptake of ^14^C-labeled acetate is significantly higher in IDH-mutant cells than in IDH-wildtype cells [84]. An initial clinical study in 28 glioma patients reported similar results for the differentiation between the IDH-mutant and IDH-wildtype genotype using the radiotracer ^18^F-fluoroethylcholine [85]. Overall, further development of these tracers is warranted, primarily when IDH inhibitors are used in clinical routine.

### Conclusions

Amino acid PET has become increasingly relevant in the clinical care of patients with gliomas. While most amino acid PET studies focused mainly on IDH-wildtype glioma patients, the available literature regarding its use in patients with IDH-mutant gliomas suggests that this technique also adds valuable clinical information for decision-making. In particular, this has been demonstrated for detecting the most malignant tumor parts, delineating glioma extent, diagnosing treatment-related changes, and assessing treatment response in IDH-mutant glioma patients.

Nevertheless, it has to be pointed out that in a considerable fraction of available studies, the neuropathological characterization of the patients’ gliomas is either based on older WHO classifications for CNS tumors (i.e., solely on histology) or is only partially histomolecularly characterized according to current classifications (i.e., according to the WHO classification for CNS tumors from 2016 or 2021). Thus, further studies in more homogenous patient groups with well-defined glioma characteristics in line with the latest 2021 WHO classification for CNS tumors, preferably in a prospective setting, are warranted.

One prospect is the specific PET imaging of IDH mutations for response assessment in patients undergoing IDH-targeted therapies. The added clinical value is related to the considerably increased specificity of these PET probes for IDH mutations. Therefore, it offers a more reliable response evaluation since FLAIR signal alterations may be unspecific for neoplastic tissue, and 2-hydroxyglutarate MR spectroscopy is highly susceptible to artifacts.
